# Anthelminthic properties of Methylene chloride-methanol (1:1) extracts of two Cameroonians medicinal plants on *Heligmosomoides bakeri* (Nematoda: Heligmosomatidea)

**DOI:** 10.1186/s12906-017-1908-8

**Published:** 2017-08-11

**Authors:** Sergine Errole Ngouateu Teufack, Gertrude NMbogning Tayo, Marc Ngangout Alidou, Jeannette Yondo, Amely Frankline Djiomene, Josué Wabo Poné, Faùily Mpoame Mbida

**Affiliations:** Research Unit of Biology and Applied Ecology, Department of Animal Biology, Faculty of Science, University of Dschang, P.O. Box 067, Dschang, Cameroon

**Keywords:** *Annona senegalensis*, *Nauclea latifolia*, *Heligmosomoides bakeri*, Additive effect

## Abstract

**Background:**

The resistance of some medico-veterinary parasite strains as well as the unavailability and toxicity of synthetic anthelminthics on humans, animals and the impacts of their residues in the environment have pushed scientists to turn to plants with anthelminthic properties. Hence, the aim of this work was to contribute to the fight against helminths of medical and veterinary importance in general, and also to clear the environment of their free living stages.

**Methods:**

Fresh eggs of *Heligmosomoides bakeri* were obtained from the faeces of experimentally infected mice. L_1_ and L_2_ larval stages were obtained after 48 and 72 h of coproculture respectively. Methylene Chloride-Methanol (1:1) extracts of *Annona senegalensis* and *Nauclea latifolia* were diluted in DMSO or Tween 80 to prepare the following concentrations: 625, 1250, 2500, 3750 and 5000 μg/ml. The effects of extract solutions were evaluated on the embryonation of eggs, egg hatching and on L_1_ and L_2_ survival after 48, 10 and 24 h of incubation. Negative controls were 1.5% DMSO, 4% Tween 80 and a mixture of these solvents. The TLC was carried out and the profiles of secondary metabolites were made.

**Results:**

Negative controls had no effect on the embryonation, eggs hatching and on larval mortality. However, it was found that, the extracts affected the free living stages of *H. bakeri* in a concentration-dependant manner. At the highest concentration (5000 μg/ml), the rate of inhibition of embryonation obtained were 20.80%, 38.15% and 84.83% for Methylene Chloride-Methanol of *Annona senegalensis* (MCM As), *Nauclea latifolia* (MCM Nl) extracts and mixture of *Annona senegalensis* and *Nauclea latifolia* (MCM As-Nl) extract respectively. For egg hatch, the inhibition rate was 16.10%, 46.24% and 87.07% for the above three extracts respectively at the same concentration of 5000 μg/ml. On L_1_ and L_2_ larval stages after 24 h of exposure to extracts, the mortality rates of 100%, 54.76% and 96.77% against 98%, 51.44% and 100% were obtained for MCM As, MCM Nl and MCM As-Nl respectively at the highest concentration. The Methylene Chloride-Methanol of *A.senegalensis*, *N. latifolia* extracts showed the presence of alkaloids except in *N. latifolia* extract, flavonoids, sterols, triterpens, tanins, polyphenols, anthraquinons, saponins and terpenoids.

**Conclusion:**

These findings suggest that, the mixture of the two plant extracts showed an additive (synergetic effect) ovicidal effect and a slight larval mortality on L_1_ as compared to the effect of MCM As extract alone. These effects were due to the presence ao secondary metabolites identifies in the plant extracts. Thus, they may be used as possible «disinfectants» for soil transmitted nematodes.

## Background

Infection by gastrointestinal helminths constitutes a serious public health problem especially in developing countries where climatic factors (heat and humidity), poverty and the poor hygienic condition influence the proliferation of disease [1–3]. In tropical areas, human helminthosis is counted among the first seven parasitic diseases registered as Neglected Tropical Diseases (NTD) [4], whereas they shackle the health of over two million of people worldwide. In Cameroon, more than 10 million people of a total of 16.1 were infested by these parasites in 2004 [5]. In infested hosts, the disease can create malnutrition, anemia, asthenia, lethargy, and anorexia which compromise human and animal health [6]. In addition, helminthosis affect growth and intellectual development and increase the vulnerability of school age children to other diseases. In livestock, these affections affect the reproduction, weight, milk and meat production. These ailments frequently cause death in heavily infested hosts, resulting in enormous economic losses [7, 8]. Actually, several methods of helminthosis control exist and the most frequently used are chemotherapy and phytotherapy. This is often done through the use of synthetic anthelminthic combined with the management of pasture [9, 10] in developed countries while it is done by medicinal plants with anthelminthic properties in developing countries [11]. Many plants are used worldwide for multiple purposes; in Cameroon for example, *Annona senegalensis* and *Nauclea latifolia* are two medicinal plants from two different families used in traditional medicine not only for many ailments but also for abdominal pain and in particular for de-worming. The aim of this study was to evaluate the ovicidal and larvicidal activities of Methylene Chloride-Methanol of *A. senegalensis* and *N. latifolia* extracts and their mixture on *Heligmosomoides bakeri* in order to assess the synergistic or antagonistic effects of the mixture in view to monitor the free living stages of nematodes and finally to prevent host infections.

## Methods

### Plant collection

All parts (roots, stem bark, fruits and leaves) of shrubs of the two selected plants were harvested from the peripheral savannas of Foumbam in the Noun Division of the West Region of Cameroon. These plant parts were transferred to the National Herbarium of Cameroon (NHC) for authentification. The plant specimens were first identified by Nnomo Laurent a botanist of the Department of Plant Biology of the Faculty of Science of the University of Dschang as *Annona senegalensis* Pers, 1934 and *Nauclea latifolia* Smith, 1919 (*Sarcocephalus latifolius* Bruce) and were registered later respectively under numbers 43,530/NHC and 47,652/NHC at the NHC. Plant stem barks of each species were dried in the shade for 1 to 6 h per day, ground to powder form and stored in separate airtight plastic bags in the Research Unit of Biology and Applied Ecology (LABEA) for further use.

### Obtention and preparation of plant extract

The procedure used to obtain the different plant extracts was according to Wabo Poné et al. [1] and D’Angelo et al. [8]. Briefly, two hundred (200) grams of the stem barks powder were macerated in 3 l of Methylene Chloride-Methanol mixture (1:1) to maximize the yield of the extraction. The mixture was kept in the laboratory for seventy two (72) hours. During this period, it was stirred daily to speed up the extraction process. It was then filtered successively through two metallic sieves (mesh sizes: 500 μm and 150 μm), a layer of cotton and filter paper (Joseph N° 1). The last filtrates were then evaporated in a rotavapor (Model Büch-R-124). The extract obtained was poured in 4 inox plates and kept in the oven at 45 °C for 24 h for full evaporation of the solvent. Each plant extract was separately diluted and mixed volume per volume at the start of the experiment. For this, two hundred (200) mg of each extract were dissolved separately in 0.8 ml of Tween 80 and 0.3 ml of DMSO (to facilitate the mixing with water). Distilled water was added to the diluted extracts in a 100 ml beaker to bring the volume up to 20 ml, thus obtaining a stock solution of 10,000 μg/ml. Through serial dilutions, concentrations of 5000, 3750, 2500, 1250 and 625 μg/ml were made. 4% Tween 80 and 1.5% DMSO were used as negative controls. Methylene Chloride-Methanol extraction yields were 9.5 and 10% for *A. senegalensis* and *N. latifolia* respectively.

### Recovery of nematode eggs and larvae

Fresh eggs of *Heligmosomoides bakeri* obtained from the faeces of experimentally infected mice were put to coproculture for 36–48 h and 72–96 h to obtain L_1_ and L_2_ larvae respectively [12].

### Evaluation of the effect of extracts on different free stages of parasite

**Fig. 1:**
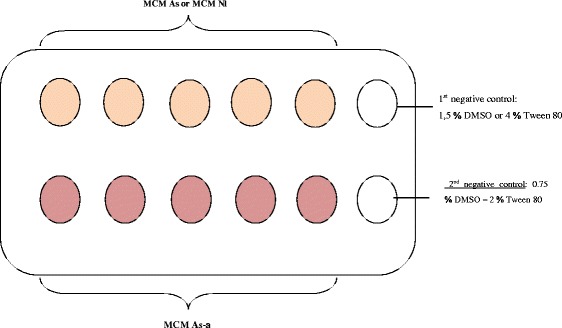
Experimental design.. Legend: MCM As = Methylene Chloride-Methanol extract of *Annona senegalensis*. MCM Nl = Methylene Chloride-Methanol extract of *Nauclea latifolia* MCM As-Nl = Methylene Chloride-Methanol mixture of extract of *Annona senegalensis* and *Nauclea latifolia.* DMSO: Dimethylsulfoxid

The methodology used in this part was the routine procedure used in the Research Unit of Biology and Applied of University of Dschang. All treatments had four replicates. For these manipulations, the experimental design proposed by Wabo Poné [11] is shows in Fig. 1.

### Evaluation of ovicidal effect of extracts

The effect of the extract on eggs was evaluated in terms of embryonation of fresh eggs and hatching of embryonated eggs.

For embryonation, 1 ml of suspension containing 30 to 40 fresh eggs was introduced in 6 Petri dishes. Thereafter, 1 ml of different concentration of single plant species was added to the Petri dishes (for the mixed extract, 0.5 ml for MCM As and 0.5 ml for MCM Nl). After 48 h of incubation time, the inhibition rate of embryonation (IRE) was estimated using the following formula (1):

1 $$ \mathrm{IRE}=100-\left[\left(\frac{\mathrm{Number}\  \mathrm{of}\  \mathrm{embryonated}\  \mathrm{eggs}}{\mathrm{Number}\  \mathrm{of}\  \mathrm{fresh}\  \mathrm{eggs}\  \mathrm{incubated}}\right)\times 100\right] $$

For the egg hatch test, the same procedure was used except that the eggs were allowed to embryonate first (24 h after the recovery) and the inhibition rate of their hatching (IRH) was evaluated 10 h later by the following formula (2):

2 $$ \mathrm{IRH}=100-\left[\left(\frac{\mathrm{Number}\  \mathrm{of}\ {\mathrm{L}}_1\ \mathrm{larvae}}{\mathrm{Number}\  \mathrm{of}\  \mathrm{embryonated}\  \mathrm{eggs}\  \mathrm{incubated}}\right)\times 100\right] $$

Hatching was stopped by added 2–3 drops of 5% Lugol when 90% of the egg hatched in control [13]. Thus, the inhibition rate of eggs hatching was fixed at 10% for negative control and treated groups.

### Evaluation of the larvicidal effect of extracts

One (1) ml of suspension containing 10 to 15 L_1_ or L_2_ larvae was introduced in 6 Petri dishes. Then, 1 ml of each concentration of extract was added in a Petri dish (for the mixed extract, 0.5 ml for MCM As and 0.5 ml for MCM Nl). Twenty four (24) hours later the larvicidal effect of the extract was evaluated. Mortality rate (MR) was then estimated by the following formula (3):

3 $$ \mathrm{MR}=100-\frac{\mathrm{Number}\  \mathrm{of}\  \mathrm{dead}\  \mathrm{larvae}}{\mathrm{Number}\  \mathrm{of}\  \mathrm{incubated}\  \mathrm{larvae}}\times 100 $$

(a larva was considered dead if it was observed immobile and with a straight body for 5 to 10 s).

### ‘qualitative phytochemical screening and thin layer chromatography (TLC) profile of extracts

Secondary metabolites contained in the extracts were determined using colorimetric methods. For the separation of different phytochemical compounds in the Methylene Chloride-Methanol (1:1) extracts of *A. snegalensis* and *N. latifolia* the extracts were spotted manually using a capillary tube on precoated silicagel G TLC plates (15 X 5 cm with 3 mm thickness). The spotted plate was put into a solvent system Ethyl Acetate- methanol- water (95: 5: 2) to detect the suitable mobile phase; 10% sulphuric acid reagent was used to identify the respective compounds.

### Statistical analysis

Inhibitory and lethal concentrations 50 (IC_50_ and LC_50_) were determined using the regression line drawn in excel according to the decimal logarithm of concentration and mortality of probits. The mean embryonation inhibition, hatching inhibition and mortality rates were also compared using the chi-square test at the *P* < 0.05 significance level.

## Results

Irrespective of the free living stage considered, the negative controls i.e. 4% Tween 80, 1.5% DMSO and 2% Tween 80–0.75% DMSO, had no effect on it. However, the different extracts at different concentrations had different effects on the fresh eggs, embryonated eggs and on L_1_ and L_2_ larval survival.

### Effect of extracts on eggs

Table 1 shows the variation of the rate of inhibition of embryonation according to different concentrations. The effects of different extracts were concentration-dependent. At the highest concentration (5000 μg/ml), the inhibition rate of embryonation of the mixture extract (MCM As-Nl) was significantly (*P* < 0.05) higher (84.83%) as compared to the effect of MCM As extract (20.8%) and MCM Nl extract (16.1%).

**Table 1:** Effect of Methylene Chloride-Methanol of *Annona senegalensis*, *Nauclea latifolia* and their mixture on fresh egg (%) according to concentrations after 48 h of exposure

	Type of extracts		
Concentrations (μg/ml)	MCM As	MCM Nl	MCM As-Nl
Controls	4.5 ^a*^	4.46^a*^	0.68^a*^
625	8 ^a*^	12.73 ^a*^	40.89^bɤ^
1250	8.1^a*^	12.82 ^a*^	48.37^bc ɤ^
2500	12.8 ^a*^	31.6^bc ɤ^	60.72^bc#^
3750	13.6 ^a*^	31.8^bc ɤ^	77.18^bcd#^
5000	20.8 ^bc*^	38.15^bc ɤ^	84.83^bcd#^

Means in the different concentrations (columns) and signs of the type of extract (rows) followed by the same letters and symbols are not significantly different at 5% probability

Table 2 shows that the rate of the inhibition of eggs hatching rises with the increasing of extract concentration. At the highest concentration (5000 μg/ml), the mixed extract produced an inhibition rate of 87.04% while the single species extracts gave lower rates [MCM As extract (38.15%) and MCM Nl extract (46.24%)].

**Table 2:** Effect of Methylene Chloride-Methanol of *Annona senegalensis*, *Nauclea latifolia* and their mixture on embryonated egg (%) according to concentrations after 10 h of exposure

	Type of extracts		
Concentrations (μg/ml)	MCM As	MCM Nl	MCM As-Nl
Control	10^a*^	10^a*^	10^a*^
625	10^a*^	12.91^a*^	66.58^b ɤ^
1250	10^a*^	15.7^a*^	64.09^b ɤ^
2500	11,5^a*^	34.11^b ɤ^	68.04^b #^
3750	15^a*^	38.6^b ɤ^	81.04^bc#^
5000	16^a*^	46.24^b ɤ^	87.04^bc#^

Means in the different concentrations (columns) and signs of the type of extract (rows) followed by the same letters and symbols are not significantly different at 5% probability

### Effect of extracts on larvae

Table 3 presents the variation of mortalities rates of L_1_ larvae according to different extract concentrations. It is observed that, mortality rates also rise with the increasing extract concentration. The effect of MCM As extract was the most efficient, producing a mortality rate of 100% against 96.77% and 54.2% for MCM As-Nl and MCM Nl respectively at the highest concentration (5000 μg/ml).

**Table 3:** Effect of Methylene Chloride-Methanol of *Annona senegalensis, Nauclea latifolia* and their mixture on L_1_ larvae mortalityrate (%) according to concentrations after 24 h of contact

	Type of extracts		
Concentrations (μg/ml)	MCM As	MCM Nl	MCM As-Nl
Control	0^a*^	0^a*^	0^a*^
625	80^bc ɤ^	0^a*^	54.23^bc ɤ^
1250	92^bc#^	0^a*^	69.82^bc ɤ^
2500	99^bc ɤ^	7.78^a*^	86.75^bc ɤ^
3750	100^bc ɤ^	18.96^b*^	89.64^bc ɤ^
5000	100^bc ɤ^	54.2^bc*^	96.77^bc ɤ^

Means in the different concentrations (columns) and signs of the type of extract (rows) followed by the same letters and symbols are not significantly different at 5% probability

As on fresh and embryonated eggs, the effect of the combined extracts was also efficient on L_2_ (Table 4). At the highest concentration (5000 μg/ml) the mortality rates obtained were 52.44%, 98% and 100% for MCM Nl, MCM As and MCM As-Nl extracts respectively.

**Table 4:** Effect of Methylene Chloride-Methanol of *Annona senegalensis, Nauclea latifolia* and their mixture on L_2_ larvae mortality rate (%) according to concentrations after 24 h of contact

	Type of extracts		
Concentrations (μg/ml)	MCM As	MCM Nl	MCM As-Nl
Control	0^a*^	0^a*^	0^a*^
625	60^b ɤ^	1.4^a*^	54.76^b ɤ^
1250	68^b ɤ^	1.04^a*^	68.08^bc ɤ^
2500	80^cd ɤ^	3.06^a*^	83.33^bcd ɤ^
3750	91^cd ɤ^	10.79^a*^	100^bcde ɤ^
5000	98^ceɤ^	51.44^b*^	100^bcde ɤ^

Means in the different concentrations (columns) and signs of the type of extract (rows) followed by the same letters and symbols are not significantly different at 5% probability

### Qualitative phytochemical screening and thin layer chromatography (TLC) profile of the extracts

Table 5 presents the secondary metabolites contained in Methylene Chloride-Methanol (1:1) extracts of *A. senegalensis* and *N. latifolia* differentiated based on their colours. All the secondary metabolites identified are present in the extracts except for alkaloids absent in MCM Nl.

**Table 5:** Secondary metabolites presents in Methylene Chloride-Methanol (1:1) extracts of *Annona senegalensis* and *Nauclea latifolia*

Secondary metabolites	Extracts	
	MCM As	MCM Nl
Flavonoids	+	+
Sterols	+	+
Alkaloids	+	+
Saponins	+	+
Triterpens	+	+
Tanins	+	+
Polyphenols	+	+
Anthraquinons	+	+
Alkaloids	+	−

Legend: + = present - = absent MCM As = Methylene Chloride-Methanol of *Annona senegalensis* MCM Nl = Methylene Chloride-Methanol of *Nauclea latifolia*

**Fig. 2:**
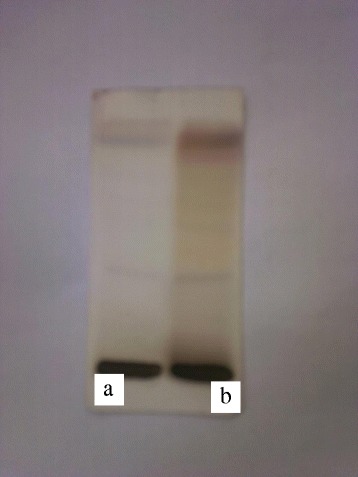
Thin Layer Chromatography (TLC) fingerprinting profile of Methylene Chloride-Methanol (1:1) extracts of barks of *Nauclea latifolia* (**a**) and *Annona senegalensis* (**b**)

The Methylene Chloride-Methanol (1:1) extracts of *A. senegalensis* and *N. latifolia*.extract were subjected to TLC, different compositions of the mobile phase were tried in order to separate the different secondary metabolites (Table 5, Fig. 2a and b). The samples were spotted on the TLC plates which were developed in the appropriate solvent system. The study revealed bioactive compounds in the stem barks extracts of *A. senegalensis* and *N. latifolia*.

## Discussion

The main advantages of using in vitro assays to test the anti-parasitic properties of plant extracts include low cost and rapid turnover which allow the screening of plants on a large scale. These tests measure the effect of anthelminthic activity directly on the processes of development, hatching and motility of parasites [14–16]. The inhibition of embryonation rates and egg hatching rates obtained in controls were very low. These results are similar to those obtained by Payne et al. [16]. According to Katiki et al. [17], DMSO and Tween 80 are two diluents mostly used for the screening of new anti-parasitic substances because these diluents are tolerated by eggs and larvae of nematode in low concentration. In general, the activity of extracts increases with the rise in concentrations. It had been proposed that an increase in concentration brings about a supplementary input of different active compounds resulting in higher effects [1].

The effect of MCM As and MCM Nl extracts were less active on embryonation and egg hatching of *H. bakeri* as compared to the effect of the mixed extract. Indeed, this is in agreement with D’Angelo et al. [8] who tested MCM As extract on the same parasite. On the other hand*,* Ngangout et al. [6] using aqueous and ethanolic extracts of *N. latifolia* and *A. senegalensis* respectively *on H. bakeri* eggs obtained contrary result. The difference might be due to the extraction diluents used and the particular proportion of bio-ovicidal compounds present in each extract. In contrast, the mixed species extract MCM As-Nl was the most efficient on egg producing inhibition rates of embryonation and egg hatching of 84.83 and 87.04% respectively. The combination of the two extracts produced significantly higher effects as compared to those of each single plant species extract. It appears that, the mixture of extract has an additive effect. This effect could be due to the interaction of bioactive compounds present in the mixture. The compounds such as flavonoids, sterols, alkaloids, saponins, triterpens, tanins, polyphenols and anthraquinons were present in MCM As and MCM Nl except alkaloids in this later. These substances act specifically on the cell membrane of eggs and proteins (collagens) of larval cuticule, changing their permeability and reducing the cholesterol level in the egg membrane [14, 15, 18, 19]. This process favours the passage of secondary metabolites inside the cell where they interfere with the mechanism of cellular mitosis by inhibiting the segmentation of blastomers in the case of fresh eggs and paralyzing the larva present in eggs in the case of embryonated eggs [20].

The effect of MCM As was more visible on larvae as compared to the activity of mixed extracts while MCM Nl extract was less active. This later result was also obtained by Yondo [21] using Methylene Chloride-Methanol extract of *Pseudospondias microcarpa* on L_1_ and L_2_ larvae of *H. bakeri.* The different activities obtained were due to the synergistic effects of bio-larvicidal compounds present in the two above mentioned extracts. In fact, alkaloids are known to be more bio-larvicidal. This justifies the stronger effect of MCM As extract. On the other hand, these alkaloids were not found in MCM Nl extract, thus its low larvicidal activity. The effect of the mixture of extracts on larvae was slightly weaker than that of MCM As extract alone. This might be due to non competitive inhibition effect of MCM Nl [22] to the bioavailability of substances contained in the extract [23]. The altered and swollen aspect of larvae cuticle after exposure to extract could be as a result of penetration of secondary metabolites through worm cuticle. After the penetration of bioactive substances in the cuticule, they can act on larvae in several ways. Firstly, they can prevent the absorption of glucose or block post synaptic receptors thus paralyzing the larvae [24]. Secondly, they may also induce the release of gamma aminobutyric acid (GABA) which blocks the transmission of nerve impulses or decoupling the phosphorylation oxydative reaction which can lead to the exhortion of the energy of the larvae [25]. Thirdly, they can link to free proteins available in the gastro-intestinal tract of larvae causing anorexia and finally the death of the worm thereafter [14, 26].

The medicinal plants are rich in secondary metabolites and among the vast array of bioactive compounds alkaloids, flavonoids, glycosides, saponins and terpenoids are in high interest. Of the several methods are available for separating plant constituents, the chromatographic procedure is the most commonly used techniques for general application**.** The present TLC studies confirmed the presence of active metabolites in the Methylene Chloride-Methanol (1:1) extracts of the study species, *A. senegalensis* and *N. latifolia.* The mobile phases used for TLC separated the bioactive compounds*.*

## Conclusion

The Methylene Chloride-Methanol of *A. senegalensis* and *N. latifolia* extract mixture showed an additive ovicidal effect whereas Methylene Chloride-Methanol extract of *A. senegalensis* was more larvicidal. The extracts may be used as soil «disinfectants» and could contribute in the disruption of the life cycle of certain soil transmitted nematodes. This report confirmed the presence of the rich variety of bioactive compounds in the plant of the study and it leads for the development of the new pharmaceuticals that address hither to unmet therapeutic needs. However, further studies are needed to confirm their efficacy (in situ) and especially to investigate their potential toxic effects.

## Abbreviations

DMSO: Dimethyl sulfoxid
IRE: Inhibition rate of embryonation
LABEA: Research Unit of Biology and Applied Ecology
MCM As: Methylene Chloride-Methanol extract of *Annona senegalensis*
MCM As-Nl: Methylene Chloride-Methanol mixture of extract of *Annona senegalensis* and *Nauclea latifolia*
MCM Nl: Methylene Chloride-Methanol extract of *Nauclea latifolia*
MR: Mortality rate
NHC: National Herbarium of Cameroon
NTD: Neglected Tropical Diseases
